# Redetermination of synthetic warwickite, Mg_3_TiO_2_(BO_3_)_2_

**DOI:** 10.1107/S1600536811002157

**Published:** 2011-01-22

**Authors:** Tetsuya Kawano, Hisanori Yamane

**Affiliations:** a Institute of Multidisciplinary Research for Advanced Materials, Tohoku University, 2-1-1 Katahira, Aoba-ku, Sendai, 980-8577, Japan

## Abstract

Single crystals of warwickite, trimagnesium titanium(IV) dioxide bis­(borate), Mg_3_TiO_2_(BO_3_)_2_, were prepared by slow cooling of the melt. The title compound is isotypic with Co_3_TiO_2_(BO_3_)_2_. In contrast to the previous refinement of warwickite [Moore & Araki (1974 ▶). *Am. Mineral.* **59**, 985–1004], that reported only isotropic atomic displacement parameters for all atoms, anisotropic displacement parameters of all atoms were refined during the current redetermination. All atoms are situated on special positions (site symmetry .*m*.). One of the two Mg sites is statistically disordered with Ti atoms (ratio 1:1), while the other is fully occupied by Mg atoms. The occupancy ratio of the Mg and Ti atoms is similar to that reported in the previous study. Metal atoms (*M*) at the Ti/Mg and Mg sites are coordinated by six O atoms in form of distorted octa­hedra. Four edge-sharing *M*O_6_ octa­hedra form *M*_4_O_18_ units, which are connected by common corners into layers parallel to (010). Adjacent layers are linked along [010] into a framework structure by sharing common edges. The B atoms are located in the triangular prismatic tunnels of the framework.

## Related literature

For the structure determination of natural warwickite, Mg_3_TiO_2_(BO_3_)_2_, see: Takéuchi *et al.* (1950 ▶); Moore & Araki (1974 ▶). For the synthesis and crystal structure analysis of Co_3_ *M*O_2_(BO_3_)_2_ (*M* = Ti, Zr), see: Utzolino & Bluhm (1995 ▶). For the synthesis of Mg_5_TiO_4_(BO_3_)_2_ and Mg_3_ZrO_2_(BO_3_)_2_, see: Konijnendijk & Blasse (1985 ▶). For the structure of Mg_5_TiO_4_(BO_3_)_2_, see: Kawano & Yamane (2010 ▶). For bond-valence-sum calculations, see: Brown & Altermatt (1985 ▶). For bond-valence parameters, see: Brese & O’Keeffe (1991 ▶). For structure standardization, see: Gelato & Parthé (1987 ▶).

## Experimental

### 

#### Crystal data

Mg_3_TiO_2_(BO_3_)_2_
*M* *_r_* = 270.45
Orthorhombic, 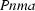

*a* = 9.3013 (5) Å
*b* = 3.10080 (14) Å
*c* = 9.3914 (6) Å
*V* = 270.86 (3) Å^3^
*Z* = 2
Mo *K*α radiation
μ = 1.94 mm^−1^
*T* = 293 K
0.17 × 0.17 × 0.12 mm

#### Data collection

Rigaku R-AXIS RAPID II diffractometer
Absorption correction: numerical (*NUMABS*; Higashi, 1999 ▶) *T*_min_ = 0.791, *T*_max_ = 0.839
2510 measured reflections
364 independent reflections
348 reflections with *I* > 2σ(*I*)
*R*_int_ = 0.018

#### Refinement

*R*[*F* ^2^ > 2σ(*F* ^2^)] = 0.026
*wR*(*F* ^2^) = 0.070
*S* = 1.20
364 reflections
44 parameters
Δρ_max_ = 0.36 e Å^−3^
Δρ_min_ = −0.59 e Å^−3^

### 

Data collection: *PROCESS-AUTO* (Rigaku/MSC, 2005 ▶); cell refinement: *PROCESS-AUTO*; data reduction: *PROCESS-AUTO*; program(s) used to solve structure: *SIR2004* (Burla *et al.*, 2005 ▶); program(s) used to refine structure: *SHELXL97* (Sheldrick, 2008 ▶); molecular graphics: *VESTA* (Momma & Izumi, 2008 ▶); software used to prepare material for publication: *SHELXL97*.

## Supplementary Material

Crystal structure: contains datablocks I, global. DOI: 10.1107/S1600536811002157/wm2443sup1.cif

Structure factors: contains datablocks I. DOI: 10.1107/S1600536811002157/wm2443Isup2.hkl

Additional supplementary materials:  crystallographic information; 3D view; checkCIF report

## Figures and Tables

**Table:** 

*M*1—O4^i^	1.9702 (12)
*M*1—O4^ii^	1.9989 (19)
*M*1—O2	2.0730 (18)
*M*1—O1^i^	2.1565 (13)
Mg2—O3^iii^	2.0043 (12)
Mg2—O4^iv^	2.0698 (19)
Mg2—O1	2.1387 (19)
Mg2—O2^v^	2.1522 (13)
B1—O3	1.353 (3)
B1—O2	1.393 (3)
B1—O1	1.395 (3)

**Table:** 

O3—B1—O2	119.1 (2)
O3—B1—O1	120.7 (2)
O2—B1—O1	120.3 (2)

Symmetry codes: (i) 
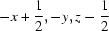
; (ii) 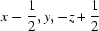; (iii) 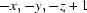; (iv) 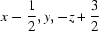; (v) 
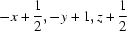
.

**Fig. 1.**
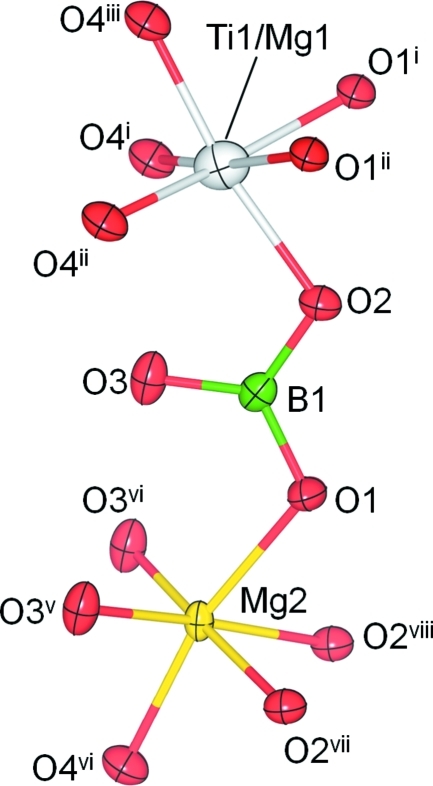
The atomic arrangement around Mg, Ti, B and O atoms in the structure of Mg3TiO2(BO3)2. Displacement ellipsoids are drawn at the 95% probability level. Symmetry codes: (i) –x + 1/2, –y, z–1/2; (ii) –x + 1/2, –y + 1, z–1/2; (iii) x–1/2, y, –z + 1/2; (iv) –x, –y, –z + 1; (v) –x, –y + 1, –z + 1; (vi) x–1/2, y, –z + 3/2; (vii) –x + 1/2, –y + 1, z + 1/2; (viii) –x + 1/2, –y, z + 1/2.

**Fig. 2.**
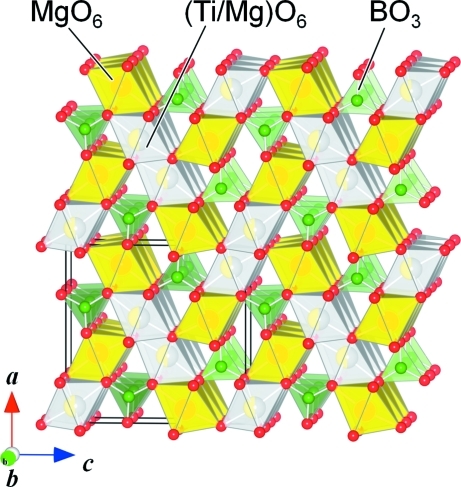
The crystal structure of Mg3TiO2(BO3)2 in a representation using cation-centred oxygen polyhedra.

## supplementary crystallographic information

### Comment

Crystal structure determinations of the mineral warwickite, Mg_3_TiO_2_(BO_3_)_2_, were reported by Takéuchi *et al.* (1950) and Moore & Araki (1974). The natural samples contained a few amount of Fe and Al. The crystal structure of synthetic Mg_3_TiO_2_(BO_3_)_2_ has not been analyzed up to now. We obtained single crystals of this compound during the preparation of Mg_5_TiO_4_(BO_3_)_2_ (Kawano & Yamane, 2010). Anisotropic atomic displacement parameters (*U_ij_*) of Mg, Ti, B and O atoms were refined in the present study. Moore & Araki (1974) refined isotropic atomic displacement parameters (*B*_iso_) of Mg, Ti, B and O atoms; neither *U* nor *B*-values were reported by Takéuchi *et al.* (1950).

Synthetic warwickite-type oxyborates with general composition *M*^II^_3_*M*^IV^O_2_(BO_3_)_2_ are known for Co_3_*M*O_2_(BO_3_)_2_ [*M* = Ti, Zr (Utzolino & Bluhm, 1995)] and Mg_3_ZrO_2_(BO_3_)_2_ (Konijnendijk & Blasse, 1985). However, only the crystal structures of Co_3_*M*O_2_(BO_3_)_2_ (*M* = Ti, Zr) were analyzed. The title compound Mg_3_TiO_2_(BO_3_)_2_ is isotypic with Co_3_*M*O_2_(BO_3_)_2_ [*M* = Ti, Zr (Utzolino & Bluhm, 1995)].

Figs. 1 and 2 show the coordination environments of the Mg, Ti, B and O atoms, and the crystal structure of Mg_3_TiO_2_(BO_3_)_2_, respectively. In the asymmetric unit, there is one Ti/Mg mixed site (*M*1) with occupancies of 0.5/0.5 and one Mg site (*M*2). Moore and Araki (1974) refined the site occupancy factors of Mg and Ti atoms at the *M*1 and *M*2 sites, ignoring Al and Fe atoms due to their similarities with the scattering profiles of Mg^2+^ and Ti^4+^, respectively. Refined occupancy factors were *M*1 = Mg_0.96 (1)_/Ti_0.04 (1)_ and *M*2 = Mg_0.62 (2)_/Ti_0.38 (2)_ and an ideal formula of Mg(Mg_0.5_Ti_0.5_)O_2_[BO_3_] was suggested (Moore & Araki, 1974). Our refinement (*M*1 = Mg1 and *M*2 = Mg_0.5_/Ti_0.5_) is consistent with the ideal formula. Although Takéuchi *et al.* (1950) reported the atomic coordinates of natural warwickite, site occupancy factors of the *M*1 and *M*2 sites were not reported.

All atoms are at special positions (*x*, 1/4, *z*), 4*c*, with site symmetries of (.*m*.). Mg and Ti atoms occupy six-coordinated oxygen-octahedral sites, forming layers composed of *M*_4_O_18_ (*M* = Ti/Mg, Mg) units. The layers are connected by edge-sharing O4 atoms of (Ti1/Mg1)O_6_ and Mg2O_6_ octahedra into a three-dimensional framework. B1 atoms are located in triangular prismatic tunnels of the framework.

Bond valence sums (BVS; Brown & Altermatt, 1985) of the Mg, Ti and B atoms were calculated with the bond valence parameters of 1.693 Å for Mg^2+^, 1.815 Å for Ti^4+^ and 1.371 Å for B^3+^ (Brese & O'Keeffe, 1991). The BVS values of the Mg2 and B1 atoms were 2.1 and 2.9, respectively. Those of the Ti1 and Mg1 atoms at the Ti1/Mg1 site were 3.22 and 2.31, respectively. The average of these value is 2.8 and close to the expected mean valence (+3) of Mg^2+^ and Ti^4+^. The B1—O distances of 1.353 (3)–1.395 (3) Å agree well with those of isotypic Co_3_*M*O_2_(BO_3_)_2_ (*M* = Ti, Zr): 1.36 (2)–1.39 (2) Å (Utzolino & Bluhm, 1995).

Warwickite-type Mg_3_TiO_2_(BO_3_)_2_ did not emit visible light under ultraviolet excitation at room temperature, while ludwigite-type Mg_5_TiO_4_(BO_3_)_2_ shows broad blue emission (435 nm) attributed to charge transfer transitions between Ti^4+^ and O^2–^ (Konijnendijk & Blasse, 1985).

### Experimental

Starting materials were powders of MgO (99.9%, Rare Metallic), TiO_2_ (99.9%, Rare Metallic) and H_3_BO_3_ (99.99%, Sigma-Aldrich). MgO was heated at 1173–1273 K for 6–12 h in air before weighing. The powders were weighed with a molar ratio of MgO: TiO_2_: H_3_BO_3_ = 5: 1: 2.7 and mixed in an agate mortar with a pestle. The mixture was pressed into a pellet, placed in a Pt boat and heated at 1623 K for 6 h in air. Heating and cooling rates were 200 and 900 K/h, respectively. About 400 mg of the sample and 100 mg of H_3_BO_3_ were weighed and mixed. The mixture in the Pt boat was heated at 1723 K for 3 h in air and cooled to room temperature at a cooling rate of 900 K/h. The obtained sample was crushed into fragments and a colourless and transparent single-crystal of about 0.12–0.17 mm was picked up under an optical microscope.

### Refinement

The crystal structures of natural warwickites were described in the space group *Pnam* (no. 62) in the previous studies (Takéuchi *et al.*, 1950; Moore & Araki, 1974). The original single-crystal X-ray diffraction data in the present study were indexed in a different setting in space group *Pmnb* and unit-cell parameters of *a* = 3.10080 (14), *b* = 9.3013 (5) and *c* = 9.3914 (6) Å. Structure parameters were eventually standardized based on the standard setting of the space group *Pnma* using the *STRUCTURE TIDY* program (Gelato & Parthé, 1987). In the final refinement, site occupation factors (s.o.f.'s) of the Ti and Mg atoms at the Ti1/Mg1 and Mg2 sites were fixed to 0.5/0.5 and 1.0, respectively, since the freely refined s.o.f.'s of the Ti and Mg atoms at the Ti1/Mg1 site were close to 1/2, and the s.o.f. of the Mg atom at the Mg2 site was about 1.0. The highest peak in the difference electron density map is 0.36 Å from O2 while the deepest hole is -0.59 Å from Ti1/Mg1.

### Crystal data

**Table:**

Mg_3_TiO_2_(BO_3_)_2_	*F*(000) = 264
*M**_r_* = 270.45	*D*_x_ = 3.316 Mg m^−^^3^
Orthorhombic, *P**n**m**a*	Mo *K*α radiation, λ = 0.71073 Å
Hall symbol: -P 2ac 2n	Cell parameters from 2288 reflections
*a* = 9.3013 (5) Å	θ = 3.1–27.5°
*b* = 3.10080 (14) Å	µ = 1.94 mm^−^^1^
*c* = 9.3914 (6) Å	*T* = 293 K
*V* = 270.86 (3) Å^3^	Block, colourless
*Z* = 2	0.17 × 0.17 × 0.12 mm

### Data collection

**Table:**

Rigaku R-AXIS RAPID II diffractometer	364 independent reflections
Radiation source: fine-focus sealed tube	348 reflections with *I* > 2σ(*I*)
graphite	*R*_int_ = 0.018
Detector resolution: 10.0 pixels mm^-1^	θ_max_ = 27.5°, θ_min_ = 3.1°
ω scans	*h* = −12→11
Absorption correction: numerical (*NUMABS*; Higashi, 1999)	*k* = −3→3
*T*_min_ = 0.791, *T*_max_ = 0.839	*l* = −12→12
2510 measured reflections	

### Refinement

**Table:**

Refinement on *F*^2^	Primary atom site location: structure-invariant direct methods
Least-squares matrix: full	Secondary atom site location: difference Fourier map
*R*[*F*^2^ > 2σ(*F*^2^)] = 0.026	*w* = 1/[σ^2^(*F*_o_^2^) + (0.0347*P*)^2^ + 0.3981*P*] where *P* = (*F*_o_^2^ + 2*F*_c_^2^)/3
*wR*(*F*^2^) = 0.070	(Δ/σ)_max_ < 0.001
*S* = 1.20	Δρ_max_ = 0.36 e Å^−^^3^
364 reflections	Δρ_min_ = −0.59 e Å^−^^3^
44 parameters	Extinction correction: *SHELXL97* (Sheldrick, 2008), Fc^*^=kFc[1+0.001xFc^2^λ^3^/sin(2θ)]^-1/4^
0 restraints	Extinction coefficient: 0.039 (7)

### Special details

**Table:**

Geometry. All e.s.d.'s (except the e.s.d. in the dihedral angle between two l.s. planes) are estimated using the full covariance matrix. The cell e.s.d.'s are taken into account individually in the estimation of e.s.d.'s in distances, angles and torsion angles; correlations between e.s.d.'s in cell parameters are only used when they are defined by crystal symmetry. An approximate (isotropic) treatment of cell e.s.d.'s is used for estimating e.s.d.'s involving l.s. planes.
Refinement. Refinement of *F*^2^ against ALL reflections. The weighted *R*-factor *wR* and goodness of fit *S* are based on *F*^2^, conventional *R*-factors *R* are based on *F*, with *F* set to zero for negative *F*^2^. The threshold expression of *F*^2^ > σ(*F*^2^) is used only for calculating *R*-factors(gt) *etc*. and is not relevant to the choice of reflections for refinement. *R*-factors based on *F*^2^ are statistically about twice as large as those based on *F*, and *R*- factors based on ALL data will be even larger.

### Fractional atomic coordinates and isotropic or equivalent isotropic displacement parameters (Å^2^)

**Table:**

	*x*	*y*	*z*	*U*_iso_*/*U*_eq_	Occ. (<1)
Ti1	0.11388 (7)	0.2500	0.07167 (7)	0.0115 (3)	0.50
Mg1	0.11388 (7)	0.2500	0.07167 (7)	0.0115 (3)	0.50
Mg2	0.10160 (8)	0.2500	0.68497 (9)	0.0055 (3)	
B1	0.1708 (3)	0.2500	0.3719 (3)	0.0061 (5)	
O1	0.24045 (19)	0.2500	0.50344 (18)	0.0088 (4)	
O2	0.25030 (18)	0.2500	0.24622 (19)	0.0078 (4)	
O3	0.0255 (2)	0.2500	0.36441 (19)	0.0100 (4)	
O4	0.5095 (2)	0.2500	0.61439 (18)	0.0100 (4)	

### Atomic displacement parameters (Å^2^)

**Table:**

	*U*^11^	*U*^22^	*U*^33^	*U*^12^	*U*^13^	*U*^23^
Ti1	0.0148 (4)	0.0079 (4)	0.0119 (4)	0.000	−0.0015 (2)	0.000
Mg1	0.0148 (4)	0.0079 (4)	0.0119 (4)	0.000	−0.0015 (2)	0.000
Mg2	0.0040 (4)	0.0045 (5)	0.0079 (4)	0.000	0.0001 (3)	0.000
B1	0.0069 (12)	0.0038 (13)	0.0077 (12)	0.000	−0.0013 (9)	0.000
O1	0.0071 (8)	0.0139 (10)	0.0054 (7)	0.000	−0.0006 (6)	0.000
O2	0.0085 (8)	0.0090 (9)	0.0058 (7)	0.000	0.0004 (6)	0.000
O3	0.0063 (9)	0.0095 (10)	0.0143 (9)	0.000	−0.0017 (7)	0.000
O4	0.0102 (9)	0.0128 (10)	0.0071 (8)	0.000	−0.0020 (6)	0.000

### Geometric parameters (Å, °)

**Table:**

Ti1—O4^i^	1.9702 (12)	Mg2—O4^x^	2.0698 (19)
Ti1—O4^ii^	1.9702 (12)	Mg2—O1	2.1387 (19)
Ti1—O4^iii^	1.9989 (19)	Mg2—O2^xi^	2.1522 (13)
Ti1—O2	2.0730 (18)	Mg2—O2^xii^	2.1522 (13)
Ti1—O1^ii^	2.1565 (13)	Mg2—Mg2^vi^	3.10080 (14)
Ti1—O1^i^	2.1565 (13)	Mg2—Mg2^vii^	3.10080 (14)
Ti1—Ti1^iv^	2.9502 (11)	Mg2—Ti1^xi^	3.2465 (9)
Ti1—Mg1^iv^	2.9502 (11)	Mg2—Mg1^xi^	3.2465 (9)
Ti1—Ti1^v^	2.9502 (11)	Mg2—Ti1^xii^	3.2465 (9)
Ti1—Mg1^v^	2.9502 (11)	Mg2—Mg1^xii^	3.2465 (9)
Ti1—Ti1^vi^	3.10080 (14)	B1—O3	1.353 (3)
Ti1—Ti1^vii^	3.10080 (14)	B1—O2	1.393 (3)
Mg2—O3^viii^	2.0043 (12)	B1—O1	1.395 (3)
Mg2—O3^ix^	2.0043 (12)		
			
O4^i^—Ti1—O4^ii^	103.80 (9)	Mg2—O1—Ti1^xi^	98.20 (6)
O4^i^—Ti1—O4^iii^	83.98 (6)	B1—O1—Ti1^xii^	123.71 (9)
O4^ii^—Ti1—O4^iii^	83.98 (6)	Mg2—O1—Ti1^xii^	98.20 (6)
O4^i^—Ti1—O2	101.28 (6)	Mg1^xi^—O1—Ti1^xii^	91.94 (7)
O4^ii^—Ti1—O2	101.28 (6)	Ti1^xi^—O1—Ti1^xii^	91.94 (7)
O4^iii^—Ti1—O2	171.31 (8)	B1—O1—Mg1^xii^	123.71 (9)
O4^i^—Ti1—O1^ii^	172.87 (6)	Mg2—O1—Mg1^xii^	98.20 (6)
O4^ii^—Ti1—O1^ii^	82.00 (5)	Mg1^xi^—O1—Mg1^xii^	91.94 (7)
O4^iii^—Ti1—O1^ii^	92.60 (6)	Ti1^xi^—O1—Mg1^xii^	91.94 (7)
O2—Ti1—O1^ii^	81.39 (6)	B1—O2—Ti1	110.19 (15)
O4^i^—Ti1—O1^i^	82.00 (5)	B1—O2—Mg2^ii^	124.49 (8)
O4^ii^—Ti1—O1^i^	172.87 (6)	Ti1—O2—Mg2^ii^	100.40 (6)
O4^iii^—Ti1—O1^i^	92.60 (6)	B1—O2—Mg2^i^	124.49 (8)
O2—Ti1—O1^i^	81.39 (6)	Ti1—O2—Mg2^i^	100.40 (6)
O1^ii^—Ti1—O1^i^	91.94 (7)	Mg2^ii^—O2—Mg2^i^	92.17 (7)
O3^viii^—Mg2—O3^ix^	101.34 (9)	B1—O3—Mg2^viii^	126.96 (6)
O3^viii^—Mg2—O4^x^	88.08 (6)	B1—O3—Mg2^ix^	126.96 (6)
O3^ix^—Mg2—O4^x^	88.08 (6)	Mg2^viii^—O3—Mg2^ix^	101.34 (9)
O3^viii^—Mg2—O1	99.89 (7)	Mg1^xii^—O4—Mg1^xi^	103.80 (9)
O3^ix^—Mg2—O1	99.89 (7)	Ti1^xii^—O4—Mg1^xi^	103.80 (9)
O4^x^—Mg2—O1	167.30 (8)	Mg1^xii^—O4—Ti1^xi^	103.80 (9)
O3^viii^—Mg2—O2^xi^	175.34 (6)	Ti1^xii^—O4—Ti1^xi^	103.80 (9)
O3^ix^—Mg2—O2^xi^	83.24 (5)	Mg1^xii^—O4—Mg1^xiii^	96.02 (6)
O4^x^—Mg2—O2^xi^	91.24 (6)	Ti1^xii^—O4—Mg1^xiii^	96.02 (6)
O1—Mg2—O2^xi^	80.01 (6)	Mg1^xi^—O4—Mg1^xiii^	96.02 (6)
O3^viii^—Mg2—O2^xii^	83.24 (5)	Ti1^xi^—O4—Mg1^xiii^	96.02 (6)
O3^ix^—Mg2—O2^xii^	175.34 (6)	Mg1^xii^—O4—Ti1^xiii^	96.02 (6)
O4^x^—Mg2—O2^xii^	91.24 (6)	Ti1^xii^—O4—Ti1^xiii^	96.02 (6)
O1—Mg2—O2^xii^	80.01 (6)	Mg1^xi^—O4—Ti1^xiii^	96.02 (6)
O2^xi^—Mg2—O2^xii^	92.17 (7)	Ti1^xi^—O4—Ti1^xiii^	96.02 (6)
O3—B1—O2	119.1 (2)	g1^xii^—O4—Mg2^xiv^	115.24 (6)
O3—B1—O1	120.7 (2)	Ti1^xii^—O4—Mg2^xiv^	115.24 (6)
O2—B1—O1	120.3 (2)	Mg1^xi^—O4—Mg2^xiv^	115.24 (6)
B1—O1—Mg2	115.18 (15)	Ti1^xi^—O4—Mg2^xiv^	115.24 (6)
B1—O1—Mg1^xi^	123.71 (9)	Mg1^xiii^—O4—Mg2^xiv^	126.50 (10)
Mg2—O1—Mg1^xi^	98.20 (6)	Ti1^xiii^—O4—Mg2^xiv^	126.50 (10)
B1—O1—Ti1^xi^	123.71 (9)		

Symmetry codes: (i) −*x*+1/2, −*y*, *z*−1/2; (ii) −*x*+1/2, −*y*+1, *z*−1/2; (iii) *x*−1/2, *y*, −*z*+1/2; (iv) −*x*, −*y*, −*z*; (v) −*x*, −*y*+1, −*z*; (vi) *x*, *y*+1, *z*; (vii) *x*, *y*−1, *z*; (viii) −*x*, −*y*, −*z*+1; (ix) −*x*, −*y*+1, −*z*+1; (x) *x*−1/2, *y*, −*z*+3/2; (xi) −*x*+1/2, −*y*+1, *z*+1/2; (xii) −*x*+1/2, −*y*, *z*+1/2; (xiii) *x*+1/2, *y*, −*z*+1/2; (xiv) *x*+1/2, *y*, −*z*+3/2.
